# Are blood lipids associated with microvascular complications among type 2 diabetes mellitus patients? A cross-sectional study in Shanghai, China

**DOI:** 10.1186/s12944-019-0970-2

**Published:** 2019-01-18

**Authors:** Hua Yang, Doris Young, Jian Gao, Yuanzhi Yuan, Minqian Shen, Yuan Zhang, Xueyan Duan, Shanzhu Zhu, Xiaoming Sun

**Affiliations:** 1 Department of General Practice, Zhongshan Hospital of Fudan University, Shanghai, China; 2 Department of General Practice, University of Melbourne, Carlton, Melbourne, VIC Australia; 3 Department of Nutrition, Zhongshan Hospital of Fudan University, Center of Clinical Epidemiology and Evidence-based Medicine, Fudan University, Shanghai, China; 4 Department of Ophthalmology, Zhongshan Hospital of Fudan University, Shanghai, China; 5 Department of General Practice, Shenzhen Longhua District Central Hospital, Shenzhen, Guangdong China; 6 Pudong Institute for Health Development, Shanghai, China

**Keywords:** Blood lipids, Diabetic kidney disease, Diabetic retinopathy, Type 2 diabetes mellitus

## Abstract

**Background:**

Although there are several studies to investigate the association between blood lipids and microvascular complications, these studies reported conflicting results. The aim of the current study was to explore the association between blood lipid parameters and the risk of microvascular complications, especially the dose-response association between them, among community patients with type 2 diabetes mellitus (T2DM) in Shanghai, China.

**Methods:**

The cross-sectional study was conducted in 6 community health service centers in Shanghai between December 2014 and December 2016.The associations between blood lipids and diabetic kidney disease (DKD) or diabetic retinopathy (DR) were assessed using multiple logistic regression. Restricted cubic spline (RCS) was employed to estimate the dose-response relation of blood lipids and the risk of microvascular complications.

**Results:**

A total of 3698 participants were included in the final analysis to study the association between blood lipids and DKD, wherein 33.2% of participants had DKD and 1374 were included for the analysis of the association between blood lipids and DR, wherein 23.2% of participants had DR. DKD odds ratio was increased by 1.16(95%CI,1.08–1.25), 1.21(95%CI,1.13–1.30), 1.18(95%CI,1.10–1.26) for comparing fourth to first quartiles of triglycerides (TG), TG/high-density lipoprotein cholesterol (HDL-C), non-HDL-C/HDL-C, respectively, and decreased by 0.83(95%CI,0.78–0.89) for comparing fourth to first quartiles of HDL-C. Furthermore, the dose-response association between TG, HDL-C, TG/HDL-C, non-HDL-C/HDL-C and the risk of DKD demonstrated turning points in TG of 1.90 mmol/L, HDL-C of 1.62 mmol/L, TG/HDL-C of 2.00, non-HDL-C/HDL-C of 3.09, respectively. However, no significant association was found between blood lipid parameters and DR.

**Conclusions:**

This community-based study indicated that TG, HDL-C, TG/HDL-C, non-HDL-C/HDL-C were independently associated with DKD but not DR.

## Background

Diabetic kidney disease (DKD) and diabetic retinopathy (DR) are major microvascular complications of type 2 diabetes mellitus (T2DM), which produces an enormous burden to national health-care systems. It has been estimated that 20 to 40% of patients with diabetes mellitus will develop DKD [1], which is the leading cause of end-stage renal disease in the Western countries [2] and ranks second in China [3], Over one-third of patients with diabetes mellitus have signs of DR [4], which is the leading cause of blindness in developed countries [5].

Several studies have demonstrated that hyperglycemia and hypertension play important roles in the development and progression of DKD and DR [6, 7]. However, other studies demonstrated that in spite of the reaching of recommended blood glucose and blood pressure targets, the risk for DKD and DR still remained high among T2DM patients [8–10]. Thus, exploring the association between microvascular complications and other metabolic elements has become increasingly important. More and more evidences suggest the involvement of dyslipidemia in the development of DKD and DR [11, 12]. Some studies have demonstrated that plasma triglycerides (TG), high-density lipoprotein cholesterol (HDL-C), and TG/HDL-C ratio, but not low-density lipoprotein cholesterol (LDL-C) were associated with microvascular complications [13–15]. Few other studies reported conflicting results. The Kidney Early Evaluation Program (KEEP) [16] demonstrated that only HDL-C was associated with urinary albumin to creatinine ratio (UACR). Sjølie AK et al. [17] found that TG, but not cholesterol was a significant risk factor for moderate-severe non-proliferative retinopathy and proliferative retinopathy. However, the association between blood lipids and microvascular complications still were poorly defined in Chinese community patients with T2DM.

Therefore, the objective of the present cross-sectional study was to explore the association, especially the dose-response association, between blood lipid parameters and the risk of microvascular complications among the target populations.

## Methods

A cross-sectional study was conducted among T2DM patients in six communities of Shanghai. The study protocol was approved by the Ethics Committee of Zhongshan Hospital of Fudan University (B2016–029).

### Population

In the current study, a stratified random sampling procedure was conducted to recruit participants from December 2014 to December 2016. Three districts were purposefully selected from sixteen districts of Shanghai, where Xuhui represented old central district, Pudong the new central and Jiading the outer fringe district, respectively. Next, we selected two community health centers (CHCs) randomly from each district, with the inclusion of six CHCs in total. Finally, we randomly recruited participants who were 18 years of age or older, diagnosed with T2DM for ≥3 months previously, incorporated into diabetes mellitus management system of China [18] from these six CHCs. Patients with pregnancy were not included in the current study. Considering the prevalence of microvascular complications in type 2 diabetes patients in previous studies [1, 4], and with a questionnaire failure rate of 20%, we required a minimum sample size of 1920 to be studied. A total of 3977 subjects were finally enrolled. Written informed consent was obtained from all the volunteers.

### Measurements

All data were obtained from field investigations, including a face-to-face interview, physical examination and biochemical measurement. The interview questionnaire contained age, gender, educational attainment, marital status, smoking status, family history of diabetes, duration of diabetes, current medical treatment, history of hypertension, coronary heart disease and stroke. Physical examination included the measurements of body weight, height, and blood pressure. Body weight and height were measured by trained investigators using standardized weight scales and meter measures. Blood pressure was measured with an arm type electronic sphygmomanometer or a manual one three times after resting for more than 10 min and then the average was taken. Biochemical measurement, including fasting blood glucose (FBG), hemoglobin A1c (HbA1c), total cholesterol (TC), TG, LDL-C, HDL-C, blood urea nitrogen (BUN), creatinine (CRE), uric acid (UA), UACR and urine analysis, were performed under a fasting condition of participants in the morning. Non-mydriatic fundus photography was obtained by using standard vision digital color fundus camera (Smartscope PRO from Optomed Oy, Oulu, Finland, or Canon CR-2 from Canon Inc.) by trained general practitioners (GPs). At least two-field retinal photographs based on macula fovea as the center were taken according to the standard protocol.

### Case definitions

Albuminuria was defined as UACR≥30 mg/g in a single random urine sample, and those whose urine analysis showed microscopic hematuria or microscopic leucocyturia were excluded. DKD was defined as either albuminuria or an estimated glomerular filtration rate (eGFR) of < 60 (ml·min^− 1^(173 m^2^)^− 1^) according to the Modification of Diet in Renal Disease [19] and the formula was as follows: eGFR (ml·min^− 1^.(173 m^2^)^− 1^) = 186 × CRE (mg/dl)^-1.154^ × age^-0.203^(× 0.742,if female). Those with albuminuria or estimated glomerular filtration rate (eGFR) < 60 (ml·min^− 1^. (173 m^2^)^− 1^) due to non-diabetic kidney disease (NDKD), determined by the confirmed medical history and records of NDKD by physicians were excluded.

DR was defined as having medical history of laser treatment for DR or being diagnosed with DR according to the International Clinical Grading Standards of Diabetic Retinopathy (2002) by ophthalmologists based on non-mydriatic fundus photography. The unqualified fundus photographs according to the standard protocol were deleted. The results were categorized into two levels: those with DR and without DR.

Hypertension was defined as either systolic blood pressure (SBP) ≥140 mmHg or diastolic blood pressure (DBP) ≥90 mmHg. Participants who received antihypertensive treatment were also included in the hypertension definition.

### Quality control

The investigators including GPs, public health physicians and nurses were trained before survey. The training was performed by two GPs of Department of General Practice of Zhongshan Hospital, Fudan University. The training courses involved the skills of interview, how to fill in the questionnaire and measure body weight, height, and blood pressure, and how to guide the participants to collect urine specimens. Quality control visits by the study team were conducted at each community to minimize the missing data.

### Statistical analysis

Statistical analysis was carried out using SPSS software, version 17.0 (SPSS Inc., Chicago USA), SAS software, version 9.2 (SAS Institute, Cary, NC), and R, version 3.3.0 (http://www.r-project.org).

Data were presented as percentages or means±standard deviations. Variables were examined between groups using Chi-square tests for the percentages and unpaired t tests for the mean values. The parameters of blood lipids, including TC, TG, LDL-C, HDL-C, Non-HDL-C, TG/HDL-C, and Non-HDL-C/HDL-C were divided into quartiles according to the linear scores. The associations between blood lipids and DKD or DR were assessed via multiple logistic regression. Multiple logistic regression models were fitted with or without DKD or DR as dependent variables and the quartiles of the blood lipid parameters as independent variables. The lowest quartile of the blood lipid parameters were used as reference in this model. Adjustments were made for potential confounders, including age, gender, smoking status, family history of diabetes, duration of diabetes and current medical treatment, comorbid hypertension, comorbid CAD, comorbid stroke, BMI, HbA1c level, and SBP. Furthermore, restricted cubic spline (RCS) was employed to estimate the dose-response relationship of blood lipids with the risk of microvascular complications [20]. Odds ratios (ORs) and 95% confidence intervals (CIs) were calculated. A two-tailed alpha with *P* < 0.05 was considered statistically significant for all analyses.

## Results

### Characteristics of participants

**Fig. 1:**
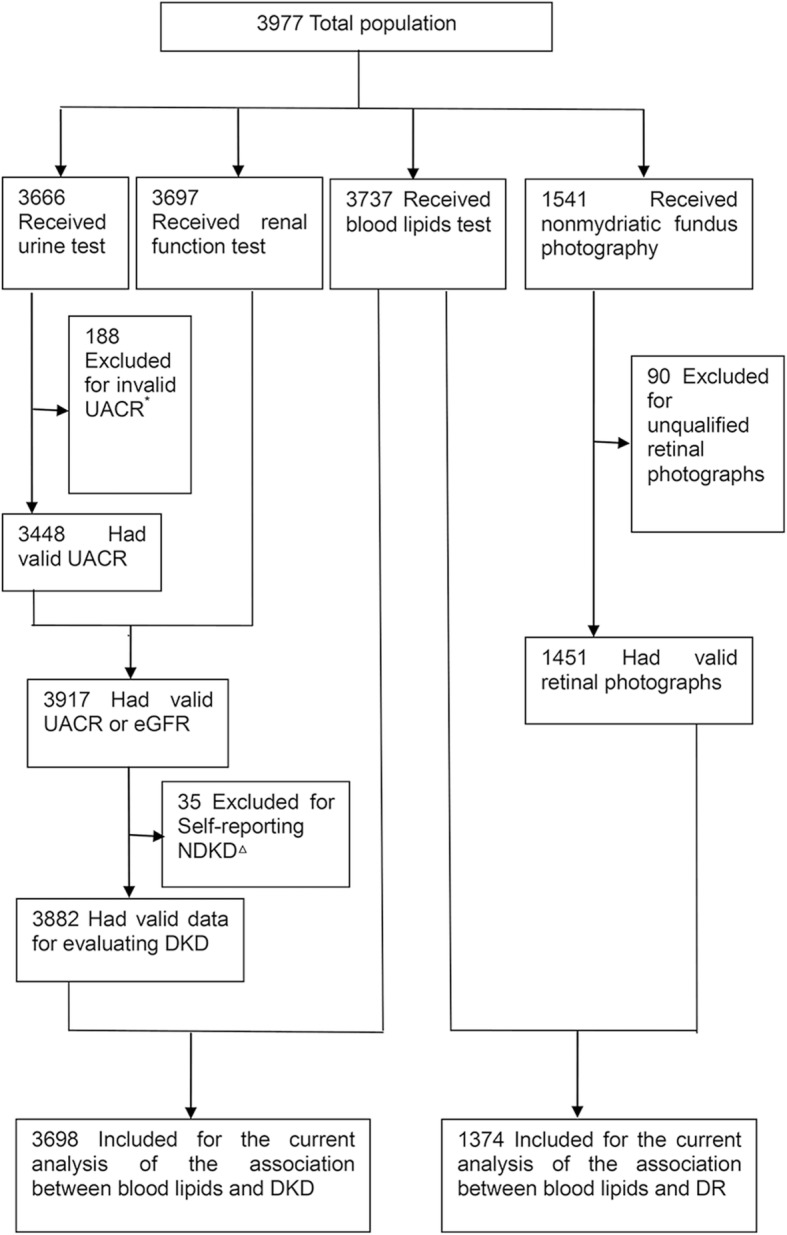
Inclusion/exclusion criteria of the study participants for assessing the association between blood lipids and microvascular complications. ^*^: Invalid UACR was defined as having microscopic hematuria or microscopic leucocyturia in urine routine. ^△^: Nondiabetic Kidney Disease (NDKD) was determined by physicians according to medical history and records of the participant

A total of 3977 participants without missing basic socio-demographic characteristics data were enrolled in this study. Of these, 3698 participants were included in the current analysis to study the association between blood lipids and DKD, and 1374 participants were included in the current analysis to study the association between blood lipids and DR (Fig. 1).

Analysis of kidney disease revealed that 33.2% of participants had DKD. Participants with DKD had older age, longer duration of diabetes, higher proportion of using insulin, having comorbid hypertension, CAD, stroke, DR, higher levels of BMI, SBP, HbA1c, TG, HDL-C, non-HDL-C, and lower level of eGFR than those without DKD (*P* < 0.05, Table 1).

**Table 1:** Socio-demographic and clinical characteristics of participants according to DKD and DR

Variable	DKD			DR		
	No (n^d^=2472)	Yes (n^d^=1226)	*P* ^e^	No (n^d^=1055)	Yes (n^d^=319)	*P* ^f^
Gender (male), n (%)	1086(43.9)	518(42.3)	0.332^h^	442(41.9)	142(44.5)	0.407 ^h^
Age (years), mean ± SD	66.8 ± 8.1	69.1 ± 8.7	< 0.001^g^	65.7 ± 7.4	64.8 ± 7.6	0.045 ^g^
Current smoker, n (%)	443(17.9)	172(14.0)	0.003 ^h^	162(15.4)	66(20.7)	0.025 ^h^
Family history of diabetes mellitus, n (%)	703(29.8)	345(29.7)	0.934 ^h^	372(37.2)	119(38.6)	0.649 ^h^
Duration of diabetes mellitus(years), mean ± SD	8.8 ± 6.1	9.8 ± 6.6	< 0.001 ^g^	8.1 ± 5.7	11.2 ± 6.6	< 0.001 ^g^
Current medical treatment, n (%)						
Without medicine	209(9.1)	96(8.4)	< 0.001 ^h^	50(5.4)	7(2.4)	< 0.001 ^h^
Oral medicine	1769(77.1)	847(73.7)		775(83.0)	210(71.2)	
Insulin	134(5.8)	113(9.8)		45(4.8)	23(7.8)	
Oral medicine + insulin	181(7.9)	93(8.1)		64(6.9)	55(18.6)	
Hypertension, n (%)	1789(72.4)	1012(82.5)	< 0.001 ^h^	795(75.4)	245(76.8)	0.598 ^h^
CAD, n (%)	253(10.2)	155(12.6)	0.028 ^h^	98(9.3)	39(12.2)	0.125 ^h^
Stroke, n (%)	189(7.6)	122(10.0)	0.017 ^h^	98(9.3)	32(10.0)	0.691 ^h^
BMI (kg/m^**2**^), mean ± SD	25.2 ± 3.2	25.7 ± 3.5	< 0.001 ^g^	25.3 ± 3.2	25.5 ± 3.2	0.460 ^g^
SBP (mmHg), mean ± SD	134.4 ± 15.1	139.0 ± 18.0	< 0.001 ^g^	137.1 ± 17.3	140.4 ± 19.0	0.004 ^g^
DBP (mmHg), mean ± SD	79.0 ± 8.3	79.1 ± 8.7	0.720 ^g^	79.1 ± 8.7	78.3 ± 9.1	0.189 ^g^
HbA1c (%), mean ± SD	7.0 ± 1.3	7.5 ± 1.7	< 0.001 ^g^	7.0 ± 1.2	7.6 ± 1.5	< 0.001 ^g^
TC (mmol/L), mean ± SD	4.7 ± 1.0	4.8 ± 1.0	0.266 ^g^	4.5 ± 1.0	4.4 ± 1.2	0.164 ^g^
TG (mmol/L), mean ± SD	1.6 ± 1.1	1.7 ± 1.1	< 0.001 ^g^	1.6 ± 1.1	1.5 ± 0.9	0.193 ^g^
LDL-C (mmol/L), mean ± SD	2.6 ± 0.9	2.6 ± 1.0	0.613 ^g^	2.7 ± 0.8	2.6 ± 0.9	0.153 ^g^
HDL-C (mmol/L), mean ± SD	1.4 ± 0.4	1.3 ± 0.4	< 0.001 ^g^	1.4 ± 0.4	1.3 ± 0.4	0.217 ^g^
TG/HDL-C, mean ± SD	1.3 ± 1.2	1.4 ± 1.2	< 0.001 ^g^	1.3 ± 1.1	1.2 ± 1.0	0.366 ^g^
Non-HDL-C (mmol/L), mean ± SD	3.3 ± 1.0	3.4 ± 1.0	0.002 ^g^	3.2 ± 1.0	3.1 ± 1.1	0.294 ^g^
Non-HDL-C/ HDL-C, mean ± SD	2.6 ± 1.1	2.8 ± 1.1	< 0.001 ^g^	2.5 ± 1.0	2.5 ± 1.0	0.883 ^g^
eGFR ^a^(ml**·**min^− 1^·(173m^2^)^− 1^), mean ± SD	102.4 ± 26.3	90.0 ± 32.2	< 0.001 ^g^	102.5 ± 30.1	105.4 ± 31.2	0.147 ^g^
UACR ^b^ (mg/g), n (%)						
<30	2092(100)	108(9.0)	< 0.001 ^h^	688(69.0)	181(59.9)	0.010 ^h^
30-300	0(0)	967(80.7)		274(27.5)	104(34.4)	
>300	0(0)	123(10.3)		35(3.5)	17(5.6)	
DR^**c**^, n (%)	190(20.8) ^d^	125(28.2)	0.003 ^h^	–	–	–

*CAD* coronary heart disease, *BMI* body mass index, *eGFR* estimated glomerular filtration rate, *UACR* urinary albumin creatinine ratio, *DR* diabetic retinopathy

^a^:eGFR was calculated using the formula of Modification of Diet in Renal Disease formula: eGFR (ml·min^-1^·(1.73m^2^)^-1^)= 186 ×CRE (mg/dl)^-1.154^ ×age^-0.203^(×0.742,if female)

^b^: UACR was measured on a single random urine sample and was calculated from urinary albumin creatinine ratio

^c^: DR was defined based on the International Clinical Grading Standards of Diabetic Retinopathy (2002) by ophthalmologist according to the retinal photographs. The results were categorized into two levels: with DR or without DR

^d^: Missing values weren’t imputed into the study database or case-wise deleted; therefore, the number of respondents included in analysis differed by variable of interest

^e^: Participants with DKD in comparison to those without DKD

^f^:Participants with DR in comparison to those without DR

^g^:using *t* test

^h^:using Chi-square test

In the retinopathy disease analysis, there were 23.2% of participants with DR. Participants with DR had younger age, longer duration of diabetes, higher proportion of smoking, using oral medicine+insulin, having positive UACR, higher levels of SBP, HbA1c than those without DR (*P* < 0.05, Table 1).

### Association between blood lipids and microvascular complications

**Fig. 2:**
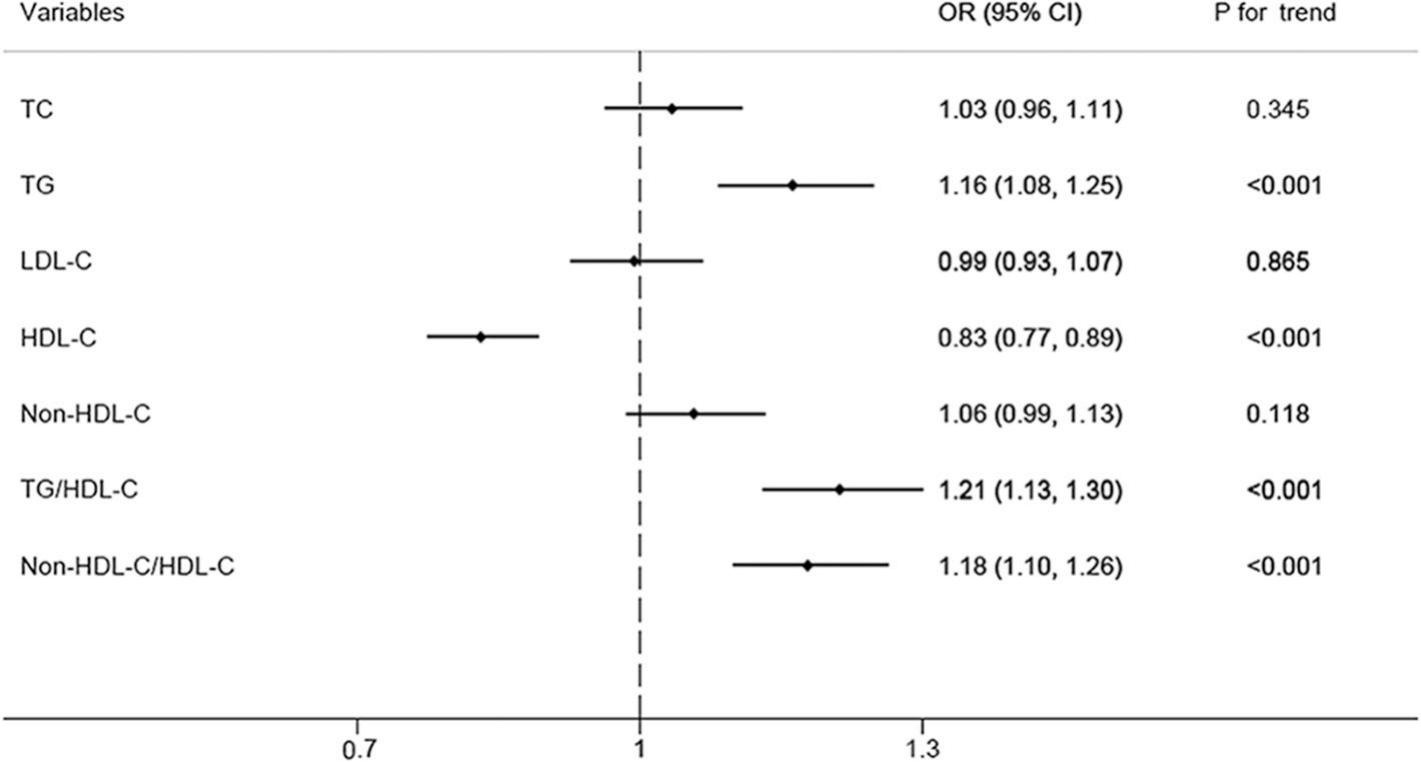
Odds ratio (OR) for participants with diabetic kidney disease, comparing fourth to first quartiles of TC, TG, LDL-C, HDL-C, Non-HDL-C, TG/HDL-C, or Non-HDL-C/HDL-C. This was performed after adjusting for age, sex, family history of diabetes mellitus, duration of diabetes mellitus, current medical treatment, smoke status, comorbid hypertension, CAD, stroke, BMI, HbA1c, SBP

T2DM participants with higher levels of TG, TG/HDL-C, non-HDL-C/HDL-C were more likely to suffer from DKD. The OR for the participants with DKD comparing fourth to first quartiles of TG, TG/HDL-C, non-HDL-C/HDL-C were 1.16(95%CI,1.08–1.25), 1.21(95%CI, 1.13–1.30), and 1.18(95%CI,1.10–1.26), respectively. T2DM participants with higher HDL-C levels were less likely to suffer from DKD. The OR for the participants with DKD comparing fourth to first quartiles of HDL-C was 0.83 (95%CI, 0.77–0.89). The trends were statistically significant for associations between TG, HDL-C, TG/HDL-C, non-HDL-C/HDL-C and the risk of DKD (*P* for trend< 0.001) (Fig. 2). This was performed after adjusting for age, gender, smoking status, family history of diabetes, duration of diabetes, current medical treatment, comorbid hypertension, CAD, stroke, BMI, HbA1c level, and SBP.

**Fig. 3:**
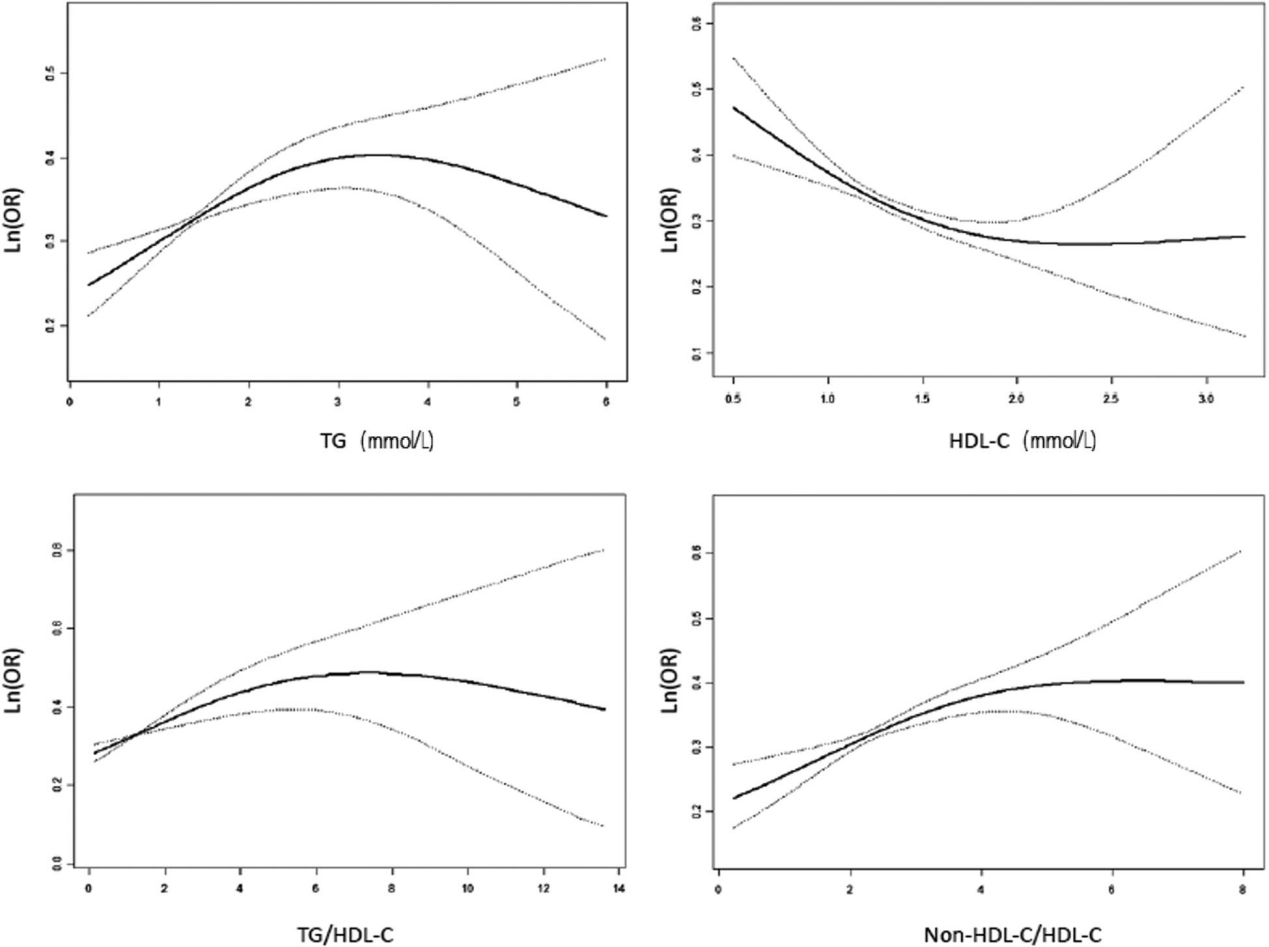
RCS on the dose-response relationship of TG, HDL-C, TG/HDL-C, Non-HDL-C/HDL-C and the risk of DKD, respectively. This was performed after adjusting for age, sex, family history of diabetes mellitus, duration of diabetes mellitus, current medical treatment, smoke status, comorbid hypertension, CAD, stroke, BMI, HbA1c, SBP. The dotted lines presented 95%CI

Furthermore, a dose-response association between TG, HDL-C, TG/HDL-C, non-HDL-C/HDL-C and the risk of DKD was found by spline analysis. When TG was lower than 1.90 mmol/L, the possibility of having DKD showed a significant increase along with increased TG levels (OR = 1.48, 95%CI, 1.21–1.81, *P* < 0.001). When TG/HDL-C was lower than 2.00, the possibility of having DKD showed a significant increase along with increased TG/HDL-C levels (OR = 1.52, 95%CI, 1.30–1.78, *P* < 0.001). When non-HDL-C/HDL-C was lower than 3.09, 3.07 in men and 2.63 in women, respectively, the possibility of having DKD showed a significant increase along with increased Non-HDL-C/HDL-C levels (OR = 1.34, 95%CI,1.17–1.53, *P* < 0.001). When HDL-C was lower than 1.62 mmol/L, the possibility of having DKD showed a significant decrease along with the increase of HDL-C levels (OR = 0.42, 95%CI, 0.31–0.58, *P* < 0.001) (Table 2, Fig. 3). Saturation effect was found when the levels of TG, HDL-C, TG/HDL-C, and non-HDL-C/HDL-C were higher than each turning point (Fig. 3). There was no interaction between gender and blood lipids effect on the risk of DKD.

**Table 2:** Dose-response association between blood lipids and the risk of DKD

Exposure Variables	TG		HDL-C		TG/HDL-C		Non-HDL-C/HDL-C	
	OR (95%CI)	*P*	OR (95%CI)	*P*	OR (95%CI)	*P*	OR (95%CI)	*P*
Total								
Turning point	1.90 mmol/L		1.62 mmol/L		2.00		3.09	
Lower than turning point	1.48(1.21–1.81)	< 0.001	0.42(0.31–0.58)	< 0.001	1.52(1.30–1.78)	< 0.001	1.34(1.17–1.53)	< 0.001
Higher than turning point	0.99(0.89–1.09)	0.793	1.68(0.95–2.95)	0.074	0.95(0.87–1.05)	0.316	0.97(0.85–1.10)	0.603
Men								
Turning point	–		1.62 mmol/L		1.96		3.07	
Lower than turning point	–	–	0.43(0.27–0.70)	0.001	1.51(1.18–1.95)	0.001	1.40(1.13–1.73)	0.002
Higher than turning point	–	–	1.15(0.41–3.21)	0.789	0.98(0.87–1.10)	0.715	1.01(0.84–1.22)	0.932
Women								
Turning point	–		1.61 mmol/L		2.09		2.63	
Lower than turning point	–	–	0.43(0.28–0.68)	< 0.001	1.59(1.29–1.94)	< 0.001	1.45(1.14–1.85)	0.003
Higher than turning point	–	–	1.72(0.87–3.39)	0.120	0.88(0.76–1.03)	0.119	0.97(0.84–1.11)	0.620

Adjusted for age, sex, family history of diabetes mellitus, duration of diabetes mellitus, current medical treatment, smoke status, comorbid hypertension, comorbid CAD, comorbid stroke, BMI, HbA1c, SBP

**Fig. 4:**
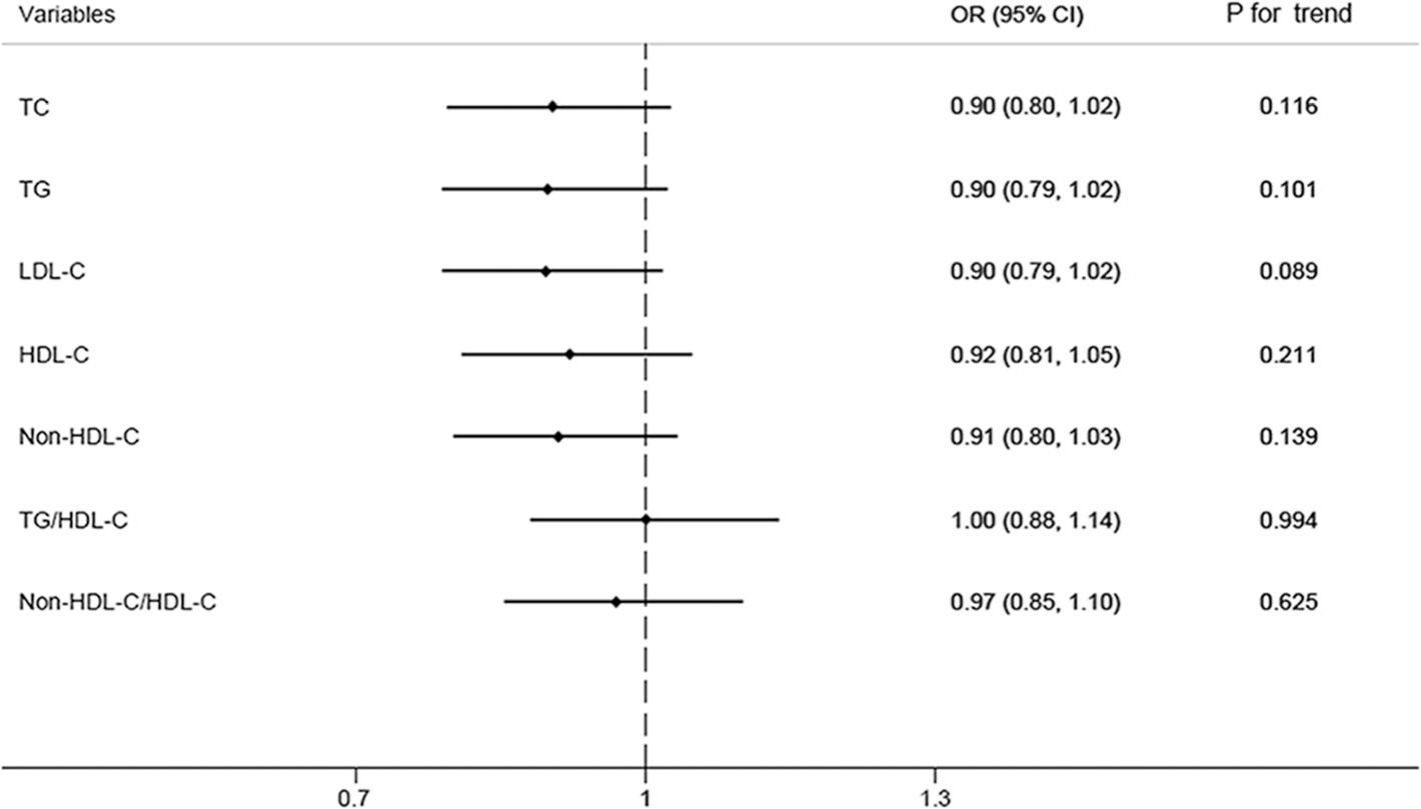
Odds ratio (OR) for participants with diabetic retinopathy comparing fourth to first quartiles of TC, TG, LDL-C, HDL-C, Non-HDL-C, TG/HDL-C, or Non-HDL-C/HDL-C. This was done by adjusting for age, sex, family history of diabetes mellitus, duration of diabetes mellitus, current medical treatment, smoke status, comorbid hypertension, CAD, stroke, BMI, HbA1c, SBP

In the current analysis, no significant association was found between blood lipid parameters and DR (Fig. 4).

## Discussion

In this current study, higher levels of TG, TG/HDL-C, non-HDL-C/HDL-C and lower levels of HDL-C, were associated with DKD, but not with DR. Notably, such associations were independent of several confounding factors, such as age, gender, smoking status, family history of diabetes, duration of diabetes, current medical treatment, hypertension, CAD, stroke, BMI, HbA1c level, and SBP. Furthermore, the dose-response association between TG, HDL-C, TG/HDL-C, non-HDL-C/HDL-C and the risk of DKD were found by RCS, which demonstrated the turning points in TG of 1.90 mmol/L, HDL-C of 1.62 mmol/L, TG/HDL-C of 2.00, and non-HDL-C/HDL-C of 3.09, respectively.

Stadler K et al. study reported the changes in serum lipid levels in T2DM and demonstrated that different blood lipids had different variation trends [21]. Most of the previous studies on the association of DKD and blood lipids demonstrated that high TG was associated with DKD [22–25], although several other studies showed no association [26, 27]. Zoppini G et al. [28] in their study followed 1987 T2DM outpatients with normal or near-normal kidney function at baseline for 5 years for the occurrence of chronic kidney disease(CKD). (CKD) They found that higher plasma levels of HDL-C were associated with lower risk of incidence of CKD in adult patients with T2DM. A large observational retrospective study of diabetic population conducted in Italy [29] demonstrated that TG ≥ 150 mg/dL increased the risk of low eGFR by 26%, albuminuria by 19%, whereas HDL-C < 40 mg/dL in men and < 50 mg/dL in women increased the risk of low eGFR by 27%, and albuminuria by 24%. Chang et al. [30] reported that the development of albuminuria was associated with higher TG and lower HDL-C levels, but not with higher LDL-C level in T2DM patients. Similar results were obtained in our current study. In addition, interesting findings of the current study include higher lipid ratios, where both TG/HDL-C and non-HDL-C/HDL-C were significantly associated with the increased possibility of having DKD. With respect to single lipid parameter usage, lipid ratios may highlight the opposite contribution of single parameters on DKD [15]. Kyung-Jin Yun et al. [31] reported that TG/HDL-C ratio may affect the development and progression of DKD in patients with T2DM and advanced DR. Zoppini G et al. [15] also found that the TG/HDL-C ratio was associated with an increased incidence of CKD in T2DM patients without prior cardiovascular disease after follow-up for 4.9 years. With regard to non-HDL-C/HDL-C ratio, some studies [32–34] showed that the ratio was probably a better predictor of metabolic syndrome, insulin resistance, arterial stiffness, and coronary heart disease. However, the relation of non-HDL-C/HDL-C ratio with DKD was not clearly presented in the previous studies. Furthermore, the dose-response association was found between TG, HDL-C, TG/HDL-C, non-HDL-C/HDL-C and the risk of DKD in the current study. When TG, TG/HDL-C, HDL-C, non-HDL-C/HDL-C were lower than each turning point, the possibility of having DKD showed significant changes along with the variations of each blood lipid levels. These findings would caution us to pay more attention to mild abnormal blood lipids levels.

Previous studies on the association between DR and blood lipids showed controversial results. Some studies [15, 35, 36] reported positive association between blood lipid parameters and DR, while others [13, 37] showed negative associations. Our study was concordant with the latter results. We showed a positive association between dyslipidemia and DKD, but negative with DR, which may be explained by different mechanisms of action of blood lipids on these two complications. Diabetic microvascular disease has complex pathogenesis, which involves endothelial dysfunction, chronic low-grade inflammation, advanced end-glycation products, oxidative stress, and abnormalities in cytokines, fibrinolysis and coagulation [38, 39]. Although the two types of microvascular diseases, DKD and DR were found to co-exist in the same patient, they were associated with different patho- physiologies.

According to the current study findings, high attention should be paid to TG, HDL-C, TG/HDL-C, and non-HDL-C/HDL-C levels in the management of DKD, especially in Chinese T2DM patients. A systematic review by Jing [40] found that the incidence of vascular complications and mortality were different in Western and Asian T2DM patients. Western patients demonstrated higher cardiovascular death rates and major coronary events, whereas Asian patients showed higher incidence of major cerebrovascular and microvascular events, including nephropathy and retinopathy. Integrated lipid targets other than LDL-C should be established to prevent and control the microvascular complications, especially DKD, in the diabetic patients.

However, our study has some limitations. (1)The blood and urine measurements were conducted by each CHC separately, but they all met the qualified standards. (2)The response rate of receiving non-mydriatic fundus photography was low, which may generate selection bias. (3)The study involved a cross-sectional design, and therefore, only association rather than causation could be evaluated. Longitudinal studies would be helpful to further explain the relationship between blood lipids and microvascular complications among T2DM patients.

## Conclusions

In conclusion, this community-based study indicated that TG, HDL-C, TG/HDL-C, non-HDL-C/HDL-C were independently associated with DKD but not with DR. The turning points in TG, HDL-C, TG/HDL-C, non-HDL-C/HDL-C associated with DKD were also found. These findings may be helpful in tailoring the screening and therapeutic strategies on microvascular complications in T2DM patients.

## Abbreviations

BUN: blood urea nitrogen
CHCs: community health centers
CIs: confidence intervals
CKD: chronic kidney disease
CRE: creatinine
DBP: diastolic blood pressure
DKD: diabetic kidney disease
DR: diabetic retinopathy
eGFR: estimated glomerular filtration rate
FBG: fasting blood glucose
GPs: general practitioners
HbA1c: hemoglobin A1c
HDL-C: high-density lipoprotein cholesterol
KEEP: Kidney Early Evaluation Program
LDL-C: low-density lipoprotein cholesterol
NDKD: non-diabetic kidney disease
ORs: odds ratios
RCS: restricted cubic spline
SBP: systolic blood pressure
T2DM: type 2 diabetes mellitus
TC: total cholesterol
TG: triglycerides
UA: uric acid
UACR: urinary albumin to creatinine ratio
